# Editorial: Immunometabolism and tumor microenvironment in hepatocellular carcinoma

**DOI:** 10.3389/fonc.2024.1483397

**Published:** 2024-09-10

**Authors:** Luyun Zhang, Lianyuan Tao, Dean Tian, Dongxiao Li

**Affiliations:** ^1^ Department of Gastroenterology, People’s Hospital of Zhengzhou University, Henan Provincial People’s Hospital, Zhengzhou, Henan, China; ^2^ Henan Key Medical Laboratory for Molecular Immunology of Digestive Diseases, Henan Provincial People’s Hospital, Zhengzhou, Henan, China; ^3^ Department of Hepatobiliary Pancreatic Surgery, People’s Hospital of Zhengzhou University, Henan Provincial People’s Hospital, Zhengzhou, Henan, China; ^4^ Henan Provincial Key Medical Laboratory for Hepatobiliary and Pancreatic Diseases, Henan Provincial People’s Hospital, Zhengzhou, Henan, China; ^5^ Department of Gastroenterology, Tongji Hospital, Tongji Medical College, Huazhong University of Science and Technology, Wuhan, China

**Keywords:** tumor microenvironment, vessel co-option, endoplasmic reticulum stress (ER stress), FOXK2, basement membrane-related genes (BMRGs), ligand-receptor interactions, UHPLC-MS

Hepatocellular carcinoma (HCC) remains a highly prevalent and lethal malignancy (1). The historically poor prognosis associated with HCC is undergoing a significant transformation due to the introduction of effective systemic therapies. Despite these advances, the molecular mechanisms underlying immune responses and evasion in HCC remain poorly understood. While the metabolic hallmarks of cancer have been extensively studied, the metabolic states of immune cells are less well characterized. Immunometabolism is an emerging field that investigates the metabolic modulation of immune cells, which is a crucial determinant of their phenotype and function within the tumor microenvironment (TME) (2). Despite remarkable progress in cancer immunotherapy, our understanding of the metabolic interactions between tumor and immune cells within the TME, as well as during immunotherapy, remains limited. Therefore, a comprehensive understanding of immunometabolism in the TME may provide innovative therapeutic perspectives for the treatment of HCC. The papers in this Research Topic illuminate the complex regulatory mechanisms that govern the interactions between tumors and the immune microenvironment. They highlight potential strategies for identifying therapeutic targets for immunotherapy, offering valuable insights into how these interactions can be leveraged to improve treatment outcomes for HCC.

Tumors can hijack existing blood vessels in the surrounding non-malignant tissue through a process known as vessel co-option (VC), which differs from neovascularization and influences disease progression, metastasis, and treatment response (3). In this Research Topic, Dan Yang et al. present a comprehensive analysis of the existing evidence, clinical outcomes, and potential molecular mechanisms associated with VC in liver cancer. Their findings indicate that VC is a significant mechanism contributing to resistance against anti-vascular therapies. Unlike vasogenic tumors, this unique vascular immune niche, characterized by distinct states and distributions of immune cells, fosters the development of an immunosuppressive tumor microenvironment that influences responses to immunotherapy. The authors further investigate the correlation between VC, drug resistance, and the immune microenvironment, offering novel insights for the advancement of liver cancer immunotherapy.

In tumors, hostile microenvironments initiate a persistent state of endoplasmic reticulum stress (ERS) that governs multiple pro-tumor properties in cancer cells while dynamically reprogramming the function of innate and adaptive immune cells (4). Consequently, Zhan et al. conducted an analysis on ERS-associated gene and identified specific gene markers associated with ERS that can effectively stratify patients into high- or low-risk groups for overall survival. The high-risk group is characterized by increased infiltration of immune cells. Furthermore, combining these ERS-associated gene markers with the tumor-node-metastasis (TNM) stage can enhance the accuracy of prognosis prediction. Notably, GP6 gene expression is down-regulated in liver cancer tissues and correlates with poor prognosis; therefore, targeted inhibition of GP6 may hold potential as a therapeutic approach.

As a member of the Forkhead box K transcription factor family, FOXK2 directly regulates the expression of genes involved in nucleotide synthesis, thereby promoting liver cancer growth and conferring chemotherapy resistance in cancer cells (5). In this context, Xing et al. provide a comprehensive overview of the current understanding of the regulatory mechanisms governing FOXK2 and its downstream targets, highlighting the significant impact that dysregulation of FOXK2 can have on cancer initiation and progression. Furthermore, they explore the potential therapeutic implications of targeting FOXK2 for tumor treatment, aiming to offer valuable insights for the diagnosis and management of conditions associated with this protein.

The basement membrane (BM) is a nanoporous matrix layer that physically separates the primary tumor from the surrounding stroma (6). Disruptions in protein expression and turnover are associated with tumor development, while the loss of basement membrane integrity is linked to tumor metastasis (7). Li et al. developed six risk score models based on basement membrane-related genes (BMRGs), revealing that a high BMRGs score correlates significantly with unfavorable prognoses in liver cancer patients, including shorter overall survival, increased extracellular matrix and signal transduction functions, and an immunosuppressive status. Additionally, two key genes, PKM and ITGA3, were found to be closely associated with poor prognosis. Thus, the BMRGs score has potential as a novel clinical predictor to enhance the prognostic evaluation of liver cancer patients.

In TME, cell communication mediated by ligand-receptor interactions orchestrates a wide array of cellular responses, including proliferation, migration, angiogenesis, immune responses, and cell death (8). Research has demonstrated that bidirectional ligand-receptor interactions at the 100 μm-wide boundaries of tumor clusters in HCC play a crucial role in maintaining the tumor’s structural integrity (9). In this context, Dai et al. identified molecular subtypes, prognostic models, and immune microenvironment characteristics based on ligand-receptor interactions involved in malignant HCC cell communication. Additionally, they elucidated the tumorigenic role of HCC cells in recruiting M2-like tumor-associated macrophages via the CCL16-CCR1 axis.

Previous clinical studies have shown that the effective rate of single-drug anti-PD-1/PD-L1 treatment is less than 20%, indicating that the majority of patients experience resistance to immunotherapy (10). This underscores the urgent need to identify biomarkers for the early prediction of immunotherapy response. In this context, Liu et al. utilized ultra-high-performance liquid chromatography-mass spectrometry (UHPLC-MS) technology to analyze the relative abundance of glycerophospholipid metabolites. Their work effectively predicts the survival benefit of anti-PD-1/PD-L1 treatment in liver cancer patients, offering a potential avenue for improving patient selection and treatment outcomes.

In summary, the editors of his Research Topic anticipate that the collection of articles will illuminate the complex interactions among the various components within TME and explore the therapeutic potential of elements that regulate the metabolic ecology of the TME. Furthermore, a comprehensive understanding of the dynamic changes in immune metabolism will facilitate the identification of potential therapeutic targets and provide a solid theoretical foundation for the development of novel immunotherapies and metabolic regulation therapies.
